# Safety and accuracy of digitally supported primary and secondary urgent care telephone triage in England: an observational study using routine data

**DOI:** 10.1186/s12911-025-02888-x

**Published:** 2025-02-03

**Authors:** Vanashree Sexton, Catherine Grimley, Jeremy Dale, Helen Atherton, Gary Abel

**Affiliations:** 1 Warwick Medical School, University of Warwick, Coventry, UK; 2 Primary Care Research Centre, University of Southampton, Southampton, UK; 3 Exeter Collaboration for Academic Primary Care, University of Exeter, Exeter, UK

**Keywords:** Digital triage, NHS 111, Primary health care, Urgent care, Emergencies

## Abstract

**Background:**

England’s urgent care telephone triage system comprises non-clinician-led primary triage (NHS111) assessment followed, for approximately 50% patients, by clinician-led secondary triage. Digital decision support is utilised by both. We explore the system’s safety and accuracy relative to patients’ use of emergency departments (EDs) and in-patient care in the subsequent 24 h.

**Methods:**

Descriptive analyses were used to investigate outcomes of 98,946 calls that underwent primary and secondary triage. We investigated sensitivity (safety) and specificity (efficiency/accuracy) in relation to subsequent ED attendance and in-patient hospital admission. Mixed effects regression models were used to explore potential under-estimation of clinical risk (under-triage).

**Results:**

Sensitivity was greater in primary triage, whilst specificity was greater in secondary triage. The positive predictive value for attending ED after being assigned a triage urgency level of within 2 h was 46.0% for secondary triage compared to 20.7% for primary triage; for inpatient admission it was 18.0% and 9.2% respectively. 1.5% (*n* = 1468) patients triaged to same-day or less urgent care at secondary triage were subsequently admitted for in-patient care. In relation to in-patient admission within 24 h, there were greater odds of potential under-triage for calls made between midnight and 6am, and for shorter duration calls, respectively OR = 1.71; CI:1.32–2.21 and OR: 1.66, CI: 1.30–2.11. The service provider (e.g., service provider 2, OR = 5.61; CI:3.36–9.36) and individual clinician (OR covering the 95% midrange = 16.15) conducting triage were the characteristics most greatly associated with this potential under-triage; *p* < 0.001 for all.

**Conclusions:**

Clinician-led urgent care triage is more accurate in identifying the likelihood of a need for ED or in-patient care than non-clinician triage. Non-clinician primary triage is risk averse, reflected in its high sensitivity but low specificity. Service and clinician characteristics associated with potential under-triage need further investigation to inform ways of improving the safety and effectiveness of urgent care telephone triage.

**Clinical trial number:**

Not applicable.

**Supplementary Information:**

The online version contains supplementary material available at 10.1186/s12911-025-02888-x.

## Background

Urgent care is routinely accessed by patients via telephone helplines. Different staff types deliver these services [1]. A non-clinician (non-medially trained operator) led model triage is widely used in the UK; whilst internationally triage is typically conducted by clinicians (usually nurses) [2], examples include Australia’s HealthDirect [3] and AskMayoClinic telephone service based in the USA [4]. Although computerised decision support is used to facilitate triage, there is limited evidence about its accuracy and safety, particularly in relation to non-clinician triage [5].

Studies have found that non-clinician triage is overly risk averse [6, 7] and may increase workload for emergency/urgent care services that are already over-burdened [8–10]. Turnbull et al. described non-clinical call handlers deferring triage decisions to clinicians [6]. Lewis et al. similarly reported risk aversion in triage, with less than 70% of patients referred to the ambulance service or advised to attend an ED subsequently attending ED [7]. In addition, potential safety issues were identified with 11% of patients advised to self-care subsequently attending ED within 48 h; of these, 88% were assessed as having urgent needs in ED with 37% subsequently being hospitalised [7].

The unique two-step telephone triage model used in most of the UK (except Northern Ireland) provides opportunity to inform how telephone-based urgent care triage can be best delivered internationally. In England, the National Health Service (NHS) 111 telephone service provides patients access to urgent care, particularly during the period outside of general practice opening hours. The service triages over 50,000 calls daily [11]. Non-clinicians (non-medically trained call operators) conduct a primary triage based on the patient’s symptoms using the NHS Pathways algorithms [12]. Approximately 24% calls are referred directly to emergency care, 8% are triaged to a primary care service such as a general practice, dental service or pharmacy, and 15% are assessed as self-care. The remaining 50% are transferred to an urgent care provider [11], and typically are called back and re-triaged (‘secondary triage’) by a clinician, usually a nurse, to categories covering the clinical urgency and the service (e.g. attending urgent care centre or emergency department (ED), home visit, routine GP appointment) needed.

Our previous study reported that 74% of NHS111 primary triage outcomes were downgraded in urgency following clinician-led secondary triage [5]. We also found that about 12% calls were upgraded during secondary triage, raising concerns about the safety of NHS111 primary triage [13]. However, no studies have compared the accuracy of primary and secondary triage as delivered within the two-step triage model. Addressing this gap is important in identifying ways of improving the efficiency and safety of urgent care, and the consequences that triage decisions have on ED and urgent care workload and waiting times. Such evidence is needed to inform health systems regarding the use of non-clinically trained staff given the possibility that it may be seen as an solution to the increasing challenges in recruiting and retaining the clinical workforce [14, 15].

Here we compare the primary and secondary triage outcomes (urgency levels) assigned to a cohort of patients who attended an ED or received hospital in-patient care in the 24-hour period following a call to the NHS111 service. It was anticipated that patients with a primary or secondary triage outcome indicating more urgent clinical needs would be more likely to attend ED or be admitted for inpatient care than patients who had less urgent triage outcomes.

### Objectives

1. To identify the frequency and characteristics of patients who attend ED or are admitted for inpatient care within 24 h post-secondary (clinician) telephone triage.
2. To describe and compare sensitivity and specificity of primary and secondary telephone triage outcomes for patients who subsequently attend ED or are admitted for inpatient care within 24 h post-secondary (clinician) triage.
3. To describe the characteristics associated with patients that were assigned a lower level of urgency at secondary triage and subsequently attended ED or were admitted for inpatient care within 24 h post-secondary (clinician) triage.

## Methods

### Study design and setting

This was an observational retrospective analysis of triage call records from three urgent care providers based in Northwest England. Call records were linked to Hospital Episode Statistics (HES) and Emergency Care Dataset (ECDS) which were provided by NHS England via data application request service (DARS: https://digital.nhs.uk/services/data-access-request-service-dars).

The participating urgent care providers each received NHS111 calls that have undergone primary triage by non-clinicians using the NHS Pathways digital triage software and been identified as having an urgent care need. They then used clinicians to conduct secondary triage supported by a different digital triage software (Odyssey); the services all had a similar skill mix in the clinicians conducting triage who are typically nurses. Further information about the digital triage software can be found in a supplementary file. We have previously described the patient population and the primary and secondary triage outcomes for this cohort [13].

### Datasets and linkage

NHS patient number and date of birth of patients were used by NHS England DARS to link datasets which were supplied as a pseudonymised dataset with all identifiable data removed.

The HES data included date/time of inpatient admission, diagnoses, and discharge date; the ECDS data included date/time of ED attendance and an acuity level (triage urgency level, that is based on a face to face triage assessment that is undertaken at the time that the patient attends the ED) [16]. The ED acuity takes one of 5 levels of urgency: 1 ‘Immediate resuscitation level care’; 2 ‘very urgent care’; 3 ‘urgent care’; 4 ‘standard care’; and 5 ‘non-urgent care’. Patients assigned to standard care are considered as having non-emergency problems suitable for treatment in an urgent care centre or minor injuries unit [16].

Dataset variables are provided in a supplementary file.

### Outcome measures

Key outcome measures were:

1. ED attendance and in-patient admission 24 h post-secondary triage.
2. ED acuity level [16].
3. Two indicators of potential under-triage (potential under-estimation of clinical risk in secondary triage) defined as: non-urgent secondary triage outcomes (care needs less urgent than “within 6 hours”) having been documented and:

#### Definition 1)

patients with ED acuity level indicative of a need for immediate or very urgent care.

#### Definition 2)

patient admitted for inpatient care 24 h post-secondary triage.

For all outcomes, a time-period of 24 h post-secondary triage was selected; during this period the patient’s use of ED or in-patient care is more likely to relate to the healthcare concern or symptoms the patient had presented with.

### Secondary triage patient records

The dataset contained 98,946 patient records of primary and secondary triage outcomes for patients who made a call to NHS111 between 1 April 2019 to 1 October 2020, triaged by 253 clinicians. As previously reported [5], this included:

- Patient, clinician and call information: call ID, anonymised patient number, anonymised clinician ID, sociodemographic characteristics, time/date of call, length of call, presenting problem.
- Primary (non-clinician) triage recommendation and secondary triage outcome recorded by a clinician. These corresponded to one of seven urgency levels: ‘emergency’; ‘immediate care within 1 h’; ‘immediate care within 2 h’; ‘urgent care within 4–6 h’; ‘same-day care within 24 h’; ‘routine primary care appointment’; and ‘self-care/no urgency’ (including advice to contact a different service). The triage urgency level selected by the clinician undertaking secondary triage was used as a proxy for disposition (care service/timeframe recommendation, e.g. advice to attend ED now), as the dataset only included the latter for a minority of calls.

### Analyses

Chi-squared tests were used to investigate differences between primary and secondary triage outcomes in relation to both the proportions of patients subsequently attending ED or being admitted and the acuity level assigned in patients that attended ED.

Safety and accuracy of primary and secondary triage outcomes were evaluated by assessing the diagnostic performance (sensitivity, specificity, positive and negative predictive values) of a given (or higher) level of triage urgency.

Two mixed effects regression models were used to explore potential under-triage (using both indicators defined in the outcomes section). Models included fixed effects: patient sex, age group, deprivation, and presenting symptom; service provider; day of week and time of day, number of calls triaged by the clinician within the full dataset (an indicator of the clinician’s familiarity with the digital triage tool) and call length. A random intercept for the clinician that conducted triage was included, enabling quantification of the variability between individual clinicians [17].

Analyses were conducted using Stata (version 18).

## Results

**Fig. 1:**
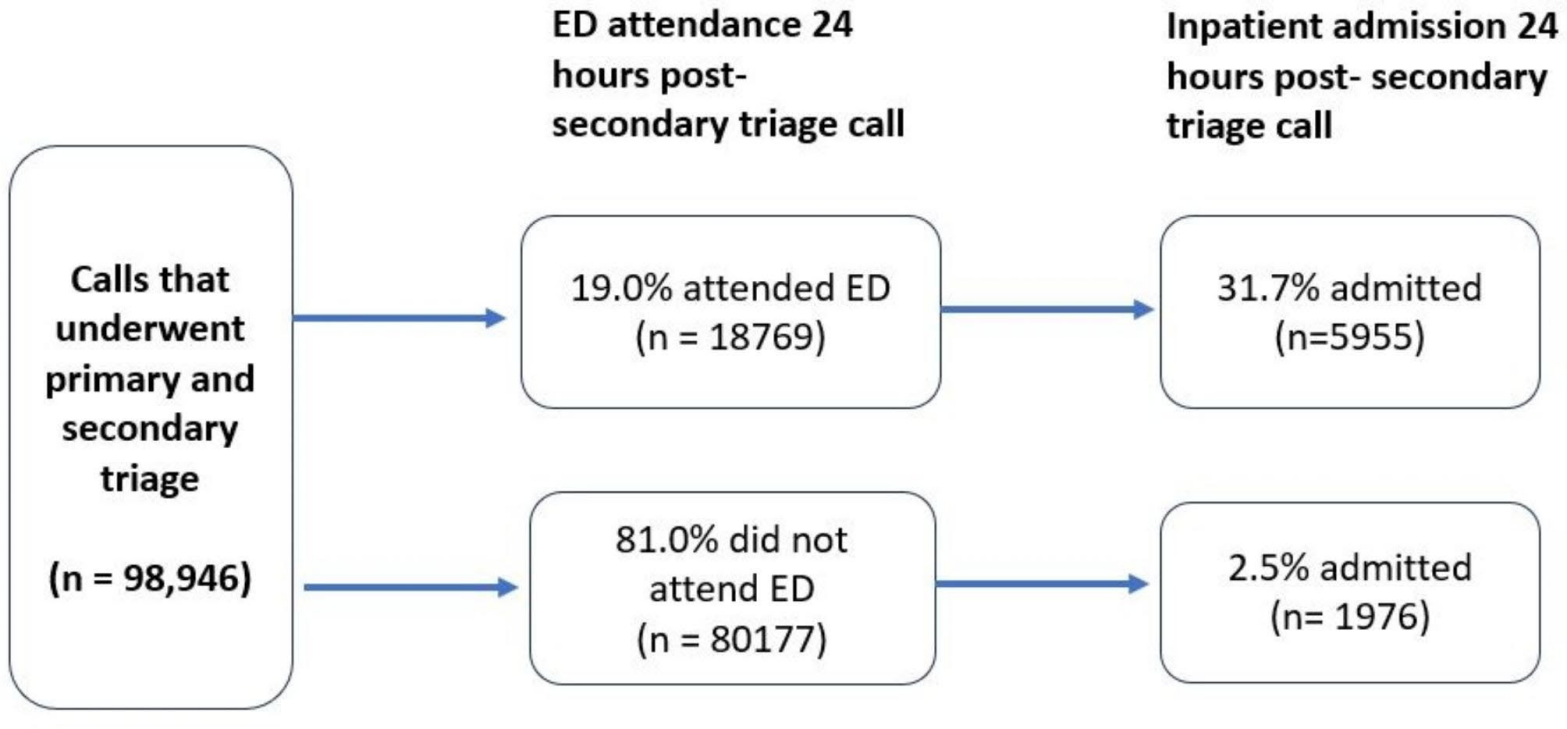
Summary of patients’ use of ED and inpatient care within 24 h post-secondary triage

In total, 20,745 (21.0%) patients attended ED and/or were admitted for inpatient care within 24 h post-secondary triage (Fig. 1). Of these, 12,814 (61.8%) only attended ED, 5955 (28.7%) were admitted to inpatient care from ED, and 1976 (9.5%) were directly admitted to inpatient care. For these patients, the most common problems that had been assessed at secondary triage were abdominal pain (*n* = 2697; 13.0%) and fever (*n* = 1360; 6.6%) (Table 1*).* Younger age groups made up a larger proportion of those attending ED and being discharged, and of those directly admitted: in children aged under 5 these were 24.2% and 38.1% of calls respectively.

In total, 59.3% (4703) patients admitted for inpatient care were discharged from hospital within one day; this comprised 3256 (54.6%) patients admitted from ED and 1447 (73.2%) patients admitted directly (without first attending ED). Characteristics of these patients are provided in supplementary file (T1).

**Table 1:** Characteristics of patients that attended ED and/or were admitted for inpatient care

	Attended ED and discharged	Attended ED and admitted	Directly admitted
**N calls (%)**	12,814 (61.8%)	5955 (28.7%)	1976 (9.5%)
**Sex**	Female *n* = 7,231 (56.4%) Male *n* = 5,583(43.6%)	Female *n* = 3,234 (54.3%) Male *n* = 2,721(45.7%)	Female *n* = 1,076 (54.5%) Male *n* = 900(45.6%)
**Age group**			
Infancy - under 24 months	*n* = 1900, 14.8%	*n* = 766,12.9%	*n* = 527, 26.7%
2–4 (young child)	*n* = 1195, 9.3%	*n* = 320,5.4%	*n* = 225, 11.4%
5–15 (child)	*n* = 1292, 10.1%	*n* = 383,6.4%	*n* = 213, 10.8%
16–24 (young adult)	*n* = 1704, 13.3%	*n* = 445,7.5%	*n* = 137, 6.9%
25–34	*n* = 2071, 16.2%	*n* = 634,10.7%	*n* = 220, 11.1%
35–44	*n* = 1172, 9.2%	*n* = 378,6.4%	*n* = 117, 5.9%
44–54	*n* = 992, 7.7%	*n* = 417,7.0%	*n* = 93, 4.7%
54–64	*n* = 858, 6.7%	*n* = 503,8.5%	*n* = 124, 6.3%
64–74	*n* = 662, 5.2%	*n* = 617,10.4%	*n* = 121, 6.1%
74–84	*n* = 590, 4.6%	*n* = 812,13.6%	*n* = 125, 6.3%
85 and over	*n* = 378, 3.0%	*n* = 680,11.4%	*n* = 74, 3.7%
**Most frequent presenting symptom**	**Abdominal Pain***n* = 1154, 9.0% **High Temperature***n* = 745, 5.8% **Chest Pain***n* = 588, 4.6% **Rash***n* = 562, 4.4% **Cough***n* = 525, 4.1% **Head Injury***n* = 448, 3.5% **Breathlessness***n* = 434, 3.4% **Vomiting***n* = 359, 2.8% **Back pain***n* = 344, 2.7% **Palpitations***n* = 248, 1.9%	**Abdominal Pain***n* = 933, 15.7% **High Temperature***n* = 379, 6.4% **Breathlessness***n* = 375, 6.3% **Cough***n* = 336, 5.6% **Vomiting***n* = 264, 4.4% **Unwell***n* = 234, 3.9% **Chest Pain***n* = 201, 3.4% **Back pain***n* = 182, 3.1% **Urinary Symptoms***n* = 172, 2.9% **Rash***n* = 156, 2.6%	**Abdominal Pain** *n* = 329, 16.7% **High Temperature***n* = 234, 11.9% **Cough***n* = 140, 7.1% **Rash***n* = 108, 5.5% **Vomiting***n* = 99, 5.0% **Breathlessness***n* = 57, 2.9% **Diarrhoea***n* = 57, 2.9% **Unwell***n* = 56, 2.8% **Back pain***n* = 47, 2.4% Urinary Symptoms *n* = 46, 2.3%

### Primary and secondary triage urgency levels in relation to ED attendance and in-patient admission

**Fig. 2:**
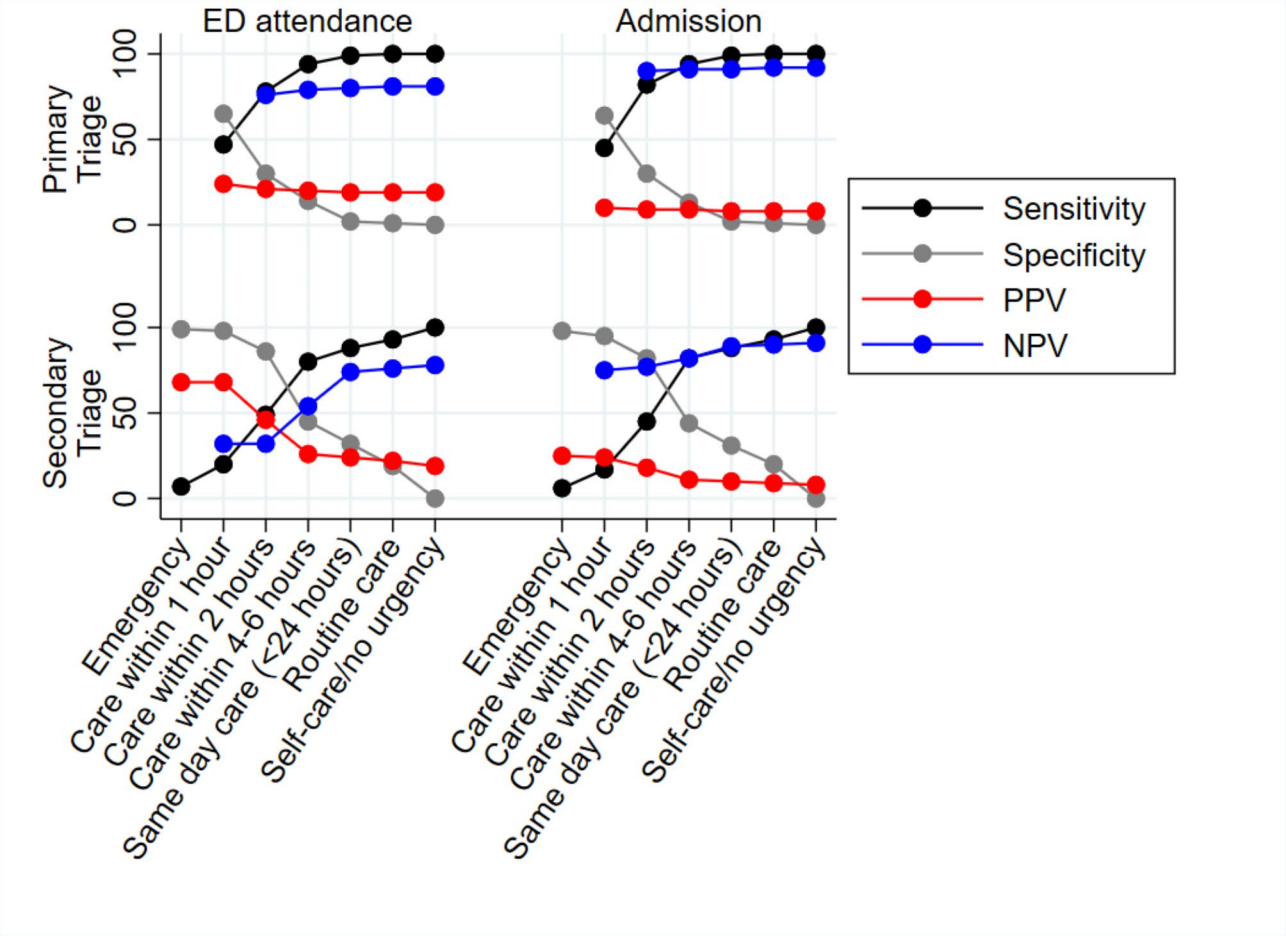
Sensitivity, specificity, and positive/negative predictive values of primary and secondary triage levels for patients attending ED within 24 h (see supplementary file T2 and T3 for underlying data)

Patients assigned a high urgency level at primary triage attended ED less frequently than those assigned equivalent urgency in secondary triage. For example, 20.8% (14616 of 70428 calls) of patients with primary triage levels indicating care needed within 2 h or less attended ED compared to 46.0% (9188 of 19984 calls) at similar urgency levels at secondary triage. Specificity (accuracy/efficiency indicator) was greater in secondary triage than primary triage at all urgency levels. For example, at care within one hour, specificity was 65.4% and 97.7%, and PPV was 24.0% and 66.7.5%, in primary and secondary triage respectively (Fig. 2, and supplementary file T2).

For in-patient admission a similar pattern was observed. For example, 9.2% (6503 of 70428 calls) of patients with primary triage levels indicating care needed within 2 h or less were admitted for inpatient care compared to 18.0% (*n* = 3592 of 19984 calls) at similar urgency levels in secondary triage. Specificity (efficiency indicator) was again greater in secondary triage than primary triage (Fig. 2, and supplementary file T3).

Sensitivity (safety/risk aversion) of primary triage was greater for both ED attendance and hospital admission than in secondary triage at all urgency levels. For example, at triage levels indicating care needed within 6 h, the sensitivity for ED attendance was 93.5% at primary triage compared to 80.4% at secondary triage (Fig. 2, and supplementary file T2), and 94.1% and 81.5% respectively for inpatient admission (Fig. 2, and supplementary file T3).

The ED-assigned acuity level was available for 86.3% (*n* = 16257) of those who attended (Table 2).

Except at the most urgent acuity levels, secondary triage urgency levels tended to be closer to those assigned in ED (Table 2). For example, for patients with an ED acuity of non-urgent or standard care, 24.6% (*n* = 1520/6182) had a secondary triage level equal to or less than same day care compared to only 7.6% (*n* = 470) for primary triage. However, for the 3003 patients assigned to immediate resuscitation or very urgent care in ED, only 698 (23.2%) had been assessed as needing care within 1 h at secondary triage compared to 1577 (52.5%) at primary triage.

**Table 2:** ED acuity assigned to patients in relation to primary and secondary triage outcomes assigned

Primary triage outcome*	Immediate resuscitation		Very urgent		Urgent		Standard		Non-urgent			Total
Emergency	n/a		n/a		n/a		n/a		n/a			
Care within 1 h	32	55.2%	1,545	52.5%	3,189	45.1%	2,687	45.4%	108	41.4%		7,561
Care within 2 h	15	25.9%	874	29.7%	2,501	35.4%	1,649	27.9%	78	29.9%		5,117
Care within 4–6 h	7	12.1%	371	12.6%	991	14.0%	1,131	19.1%	55	21.1%		2,555
Same day care (24 h)	3	5.2%	142	4.8%	359	5.1%	408	6.9%	14	5.4%		926
Routine care	1	1.7%	6	0.2%	10	0.1%	19	0.3%	3	1.1%		39
Self-care/no urgency	0	0.0%	7	0.2%	22	0.3%	27	0.5%	3	1.1%		59
**Total**	**58**		**2**,**945**		**7**,**072**		**5**,**921**		**261**			**16**,**257**
**%**	**0.4%**		**18.1%**		**43.5%**		**36.4%**		**1.6%**			
**Secondary triage outcome** **												
Emergency	5	8.6%	246	8.4%	556	7.9%	281	4.7%	2	0.8%		1,090
Care within 1 h	4	6.9%	443	15.1%	912	12.9%	629	10.6%	16	6.1%		2,004
Care within 2 h	19	32.8%	938	31.9%	2,113	29.9%	1,657	28.0%	64	24.5%		4,791
Care within 4–6 h	20	34.5%	833	28.3%	2,277	32.2%	1,925	32.5%	86	33.0%		5,141
Same day care (24 h)	2	3.4%	154	5.2%	460	6.5%	536	9.1%	31	11.9%		1,183
Routine care	5	8.6%	130	4.4%	316	4.5%	362	6.1%	31	11.9%		844
Self-care/no urgency	3	5.2%	199	6.8%	431	6.1%	529	8.9%	31	11.9%		1,193
**Total**	**58**	**100.0%**	**2**,**943**		**7**,**065**		**5**,**919**		**261**			**16**,**246**
**%**		**0.4%**	**18.1%**		**43.5%**		**36.4%**		**1.6%**			

*P value < 0.001 for differences in percentages assigned at each ED acuity level, across primary triage urgency levels (chi squared test for proportions)

**P value < 0.001 for differences in percentages assigned at each ED acuity level, across secondary triage urgency levels (chi squared test for proportions)

### Exploring potential under-triage

1.7% calls (*n* = 1725/98946) were identified as meeting one or both definitions of potential-under-triage.

#### ED attendance

Of patients attending ED, 3003 (18.5%) were assigned to ‘very urgent’ or ‘immediate resuscitation’ level care on arrival in the ED, and of these 16.4% (*n* = 493) patients met the criteria of potential under-triage having been assigned a level less urgent than “within 6 hours” in secondary triage (definition 1, described in Methods).

Of these 493 potentially under-triaged patients, 50.9% (*n* = 251) were female; they were disproportionately in the youngest age groups with 36.5% (*n* = 180) related to children under 5; the most frequent presenting symptoms were high temperature (*n* = 81,15.9%), cough (*n* = 53,10.3%) and abdominal pain (*n* = 44,8.5%); and almost half (*n* = 246; 49.9%) were admitted for in-patient care. See supplementary tables (T4-T6) for primary and secondary outcomes assigned to these calls and patient characteristics.

#### Inpatient admission

Of 7931 calls where the patient was admitted for in-patient care, 1477 (18.6%) met the criteria for potential under-triage, having had a non-urgent secondary triage outcome (care needs less urgent than “within 6 hours”). 28.2% (*n* = 417) of these calls were about children aged under 5. The top 3 symptoms called about were abdominal pain (13.5%, *n* = 199), high temperature (10.0%, *n* = 147) and cough (6.6%, *n* = 97). Patient characteristics are shown in supplementary tables (T7-T9). For patients in the potentially under-triaged sub-cohort that were admitted to inpatient care, 63.4% (*n* = 937) were discharged within 1 day of admission; of these 7.0% (*n* = 66) were recorded as elective admissions.

### Factors associated with potential under-triage

#### Patient factors

Greatest odds of potential under-triage were seen in patients with a main symptom of high temperature (model 1), OR 1.66 (1.01–2.73), *p* = 0.02; and diarrhoea (model 2), OR 1.81 (CI: 1.18–2.76), *p* < 0.001; reference symptom: abdominal pain. No other patient characteristics were associated with greater or lower odds of under-triage.

#### Call-related factors

Regression model 1 showed lower odds of potential under-triage (high urgency level assigned in ED, for calls that were assessed as low urgency in secondary triage) on weekend days.

Model 2 (potential under-triage, based on in-patient admission within 24 h of the call) indicated greater odds of under-triage for calls undergoing secondary triage in the early morning period (Midnight – 6am): OR = 1.71;CI:1.32–2.21 and in the shortest calls of 0–5 min in duration: OR: 1.66,CI:1.30–2.11, reference: 10–15 min duration; *p* < 0.001 for all.

#### Service and clinician level factors

There was service-specific variation associated with where patients were triaged. For example, compared to Service 3, the odds of potential under-triage at Service 2 were 7.14 (CI:3.74–13.63,*p* < 0.001) for definition 1 and 5.61 (CI:3.36–9.36,*p* < 0.001) for definition 2. Even greater variation was seen in relation to the individual clinician that conducted the triage call; the ORs covering the 95% mid-range of clinicians was 7.80 for potential under-triage definition 1, and 16.00 for potential under-triage definition 2.

Regression modelling results are shown in Table 3.

**Table 3:** Factors associated with potential under-triage: mixed effects regression modelling results

	Model 1: Odds of potential under-triage, where secondary triage > = care within 24 h selected, and patient subsequently assessed as requiring immediate or very urgent emergency care in ED				Model 2: Odds of potential under-triage, where secondary triage > = care within 24 h selected in patients subsequently admitted for inpatient care		
	**OR (95% CI)**	**Joint** *p* **-value**			**OR (95% CI)**	**Joint** *p* **-value**	
**Sex**							
Male	1.09 (0.87–1.35)	0.455			0.98 (0.86–1.12)	0.784	
Female	Ref				ref		
**Main presenting symptom**				**Main presenting symptom**			
Abdominal pain	Ref			Abdominal pain	ref		
High Temperature	1.66 (1.01–2.73)			High Temperature	1.25 (0.93–1.68)		
Cough	1.51 (0.90–2.53)			Cough	1.33 (0.97–1.82)		
Breathlessness	0.90 (0.52–1.55)			Vomiting	0.95 (0.68–1.35)		
Rash	0.90 (0.49–1.67)			Rash	1.24 (0.84–1.82)		
Chest Pain	0.71 (0.37–1.36)			Breathlessness	0.58 (0.40–0.84)		
Vomiting	0.99 (0.50–1.98)	0.020		Unwell	1.10 (0.74–1.64)	< 0.001	
Unwell	1.25 (0.58–2.70)			Urinary Symptoms	1.49 (1.00–2.23)		
Palpitations	0.57 (0.23–1.41)			Diarrhoea	1.81 (1.18–2.76)		
Back pain	1.06 (0.43–2.60)			Back pain	1.32 (0.87–1.99)		
Other	1.41 (0.94–2.11)			Other	1.33 (1.10–1.62)		
**IMD Decile**							
1 (most deprived)	0.75 (0.47–1.22)				0.78 (0.58–1.03)		
2	0.81 (0.48–1.37)				0.81 (0.60–1.10)		
3	0.82 (0.48–1.39)				0.78 (0.57–1.07)		
4	0.77 (0.44–1.34)				0.88 (0.63–1.23)		
5	0.65 (0.35–1.19)	0.968			0.89 (0.63–1.26)	0.601	
6	Ref				ref		
7	0.67 (0.34–1.33)				0.79 (0.54–1.13)		
8	0.71 (0.37–1.37)				0.92 (0.65–1.30)		
9	0.72 (0.35–1.46)				1.03 (0.71–1.50)		
10 (least deprived)	0.85 (0.40–1.77)				0.88 (0.58–1.34)		
**Age Group**							
Infancy - under 24 months	1.14 (0.67–1.92)				1.19 (0.88–1.61)		
2–4 (young child)	1.48 (0.86–2.55)				1.16 (0.82–1.64)		
5–15 (child)	1.19 (0.68–2.08)				1.07 (0.77–1.50)		
16–24 (young adult)	1.32 (0.75–2.33)				0.90 (0.65–1.26)		
25–34	0.77 (0.44–1.36)				0.87 (0.64–1.19)		
35–44	1.27 (0.69–2.33)	0.1419			1.03 (0.73–1.46)	0.0169	
45–54	Ref				ref		
55–64	0.96 (0.53–1.73)				0.79 (0.56–1.10)		
65–74	0.85 (0.46–1.55)				0.89 (0.65–1.23)		
75–84	0.71 (0.39–1.30)				0.72 (0.53–0.99)		
85 and over	0.83 (0.44–1.54)				0.76 (0.55–1.06)		
**Service**							
Service 1	3.21 (2.09–4.93)				2.45 (1.76–3.42)		
Service 2	7.14 (3.74–13.63)	< 0.001			5.61 (3.36–9.36)	< 0.001	
Service 3	Ref				ref		
**Day of Week**							
Sunday	0.60 (0.40–0.90)				0.94 (0.73–1.22)		
Monday	0.74 (0.46–1.19)				1.44 (1.08–1.93)		
Tuesday	1.22 (0.76–1.95)				1.37 (1.02–1.85)		
Wednesday	Ref	0.008			ref	0.009	
Thursday	1.07 (0.68–1.67)				1.27 (0.95–1.70)		
Friday	0.68 (0.42–1.10)				1.14 (0.85–1.54)		
Saturday	0.63 (0.42–0.95)				1.03 (0.80–1.33)		
**Call Time Period**							
24:00–06:00	1.35 (0.89–2.05)				1.71 (1.32–2.21)		
06:00–12:00	1.30 (0.91–1.85)	0.018			1.39 (1.13–1.70)	< 0.001	
12:00–18:00	Ref				ref		
18:00–24:00	0.86 (0.62–1.21)				1.16 (0.95–1.42)		
**Call minute category**							
0–5 min	1.01 (0.67–1.53)				1.66 (1.30–2.11)		
5–10 min	1.00 (0.74–1.33)				1.01 (0.85–1.20)		
10–15 min	Ref	0.208			ref	< 0.001	
15–20 min	0.96 (0.68–1.35)				0.97 (0.80–1.19)		
Over 20 min	0.67 (0.47–0.96)				0.83 (0.67–1.04)		
**Calls by user category**							
Under 200	0.97 (0.45–2.10)				1.22 (0.74–2.00)		
200–1000	1.20 (0.79–1.82)				0.92 (0.63–1.34)		
1000–1800	Ref	0.060			ref	0.107	
1800–2500	1.36 (0.84–2.19)				1.25 (0.79–1.98)		
2500+	2.36 (1.33–4.19)				1.73 (1.04–2.88)		
**Clinician conducting triage**	7.72				16.15		

## Discussion

This is the first study to report on the safety and accuracy of triage assessments in a two-step system of urgent care telephone triage by comparing the urgency levels assigned to patients along the care pathway with the subsequent care that they received. In total, 21% of patients attended ED and/or were admitted to hospital following two-step triage, and almost a third of these were children aged under 5 years. However, over two-thirds of ED attenders were discharged home, and it is likely that many of them had conditions that were more appropriate to being treated in urgent/primary care. Likewise, almost two-thirds of those admitted for inpatient care were discharged within a day. The specificity and predictive value of secondary triage urgency levels for ED attendance or in-patient admission was much greater than for the equivalent levels assigned in primary triage. Greater sensitivity was seen in primary triage, reflecting risk aversion [5].

There was some indication of potential under-triage in secondary triage. Applying our definitions for ‘potential under-triage’ led to a total of 1.7% (*n* = 1725/98946) being identified. This included 0.5% (*n* = 493/98946) patients in relation to their ED acuity status and 1.5% (*n* = 1468/98946) in relation to in-patient care within 24 h. Of the latter 1468 patients, a large proportion (63%) were discharged within 1 day of admission; this included some who had been admitted as day-cases for elective care.

While there were some clinical conditions (e.g., abdominal pain, fever, cough), age groups (children under of 5 years) and times of day and week when potential under-triage was slightly more likely, the service provider and individual clinician that conducted triage emerged as the key factors associated with potential under-triage despite all services having similar skillmix. There was some indication that clinicians who had shorter duration calls had greater odds of potential under-triage.

### Comparison to other literature

Our findings highlight the variation that exists between individual clinicians and services, even when they are all using the same decision support systems. This has also been described in relation to ED triage decision-making [18, 19]. While this may relate to differences in the patient populations served, and the effect this has on diagnostic accuracy [20], differences in staff training and supervision are recognised as being important [21]. Hence, while being an important indicator of clinical need, the acuity level assigned in ED is not an absolute measure of the clinical needs of the patient as it also reflects subjectivity and contextual factors.

Our study highlights poor specificity in primary triage that may contribute to increased demand on emergency care; other studies have similarly highlighted risk aversion in non-clinician triage [6, 7]. Low specificity was also described for a newly introduced non-clinician triage helpline in detecting patients who could be managed by a GP, based on a comparison of the triage advice provided by a non-clinician to a GP assessment for patients who were waiting to be seen in ED or at an urgent care centre [22]. However, due to differing settings, study designs, triage scales and patient populations, any comparison of sensitivity and specificity with other triage systems needs to be undertaken cautiously.

Other studies have compared triage assessment decision making between different types of clinicians. For example, there is some evidence that nurses are less likely to under-triage (i.e. have greater sensitivity) but more likely to over-triage (i.e. have lower specificity) (being less efficient in terms of patients’ healthcare service use) compared to GPs [23]. Likewise, over-triage has been reported to be greater in self-triage as compared to nurse triage [24]. These patterns seen in the shift from GP to nurses, or from nurses to self-triage in the ED appear to be reflected in this study: in English urgent care, non-clinicians are less likely to under-triage but more likely to be risk-averse.

### Strengths and limitations

This is the first study to report on patients’ use of ED and in-patient admissions following two-step triage, and the first to compare the sensitivity and specificity of triage outcomes against these outcomes. It is set in England, where healthcare is free at the point of access, and this may affect the transferability of the findings to other health systems.

We only had access to complete data on the urgency level of the secondary triage, and not the referral or appointment booking associated with the clinician’s triage assessment. Hence, while it is likely that all patients triaged as having an emergency will have been referred to the ED directly, for patients triaged to lower levels of urgency most will have been offered in-person consultation appointments with the urgent care service or with their own general practice. The pathway to attending ED or to inpatient admission is likely to have been varied; in many instances, it may have been appropriate for the patient to be first be seen for further assessment and physical examination by the urgent care provider before being advised to attend the hospital. In other instances, the patient’s condition may have worsened in the hours following secondary triage, and the decision to attend ED may have been in keeping with the advice given by the clinician about what to do should this occur. In addition, some patients may have attended ED or received inpatient care for clinical needs that were entirely separate to the problem that they had presented to urgent care.

The extent to which the differences in the sensitivity and specificity of primary and secondary triage outcomes relate to the specific decision support systems being deployed by care providers is important to consider. Other decision support systems may differ in accuracy and safety compared to the systems we investigated. However, a strength was that the dataset allowed comparison between the outcomes for three urgent care providers and for individual clinicians working for these services which allowed recognition of how service and user factors may affect the triage of patients. The variability identified highlights how contextual factors (e.g. differences in workforce, training and supervision, workload) may influence decision-making and the way that digital triage tools are used and hence the safety and accuracy or urgent care triage. The triage outcome urgency level recommended at the end of a secondary triage assessment is dependent on the extent to which clinicians have completed the symptom questions included in the digital triage system; it was noteworthy that in short duration calls (where question prompts may have been left incomplete) there was a higher risk of potential under-triage. It is also possible that the urgency level recorded within the digital triage software does not always reflect the actual advice that the patient was given [25].

In relation to the concept of ‘potential under-triage’ it is important to recognise the uncertainty that exists in many acute presentations; it cannot be assumed that the condition of the patient when they attended ED or were admitted for inpatient care was the same as when they first contacted urgent care or underwent secondary triage. Patients’ conditions worsen or improve in unpredictable ways over time. Additionally, the urgency assessment when the patient arrives at the ED is not standardised, and it may under- or over-estimate the urgency of the patient’s needs and is recognised as varying between individuals and EDs undertaking such assessments [18, 19].

Much of the data collected spanned the Covid-19 pandemic, and so is likely to have included greater numbers of patients presenting with symptoms associated with Covid (e.g. cough, fever, breathlessness). During the Covid-19 pandemic there were high workload pressures on health services [26, 27], and in England, media messaging to reduce demand and “protect” the NHS [28]. This may have impacted on triage decision making, influenced by both patient and professional factors [29, 30]. Finally, our definition of ‘potential under-triage’ was based on a 24-hour time interval following secondary triage; this may have excluded some patients who attended ED or were admitted to inpatient care at a later point.

### Implications for research and practice

While the overall performance of the two-step urgent care triage model deployed in England has a high degree of safety and accuracy, our findings provide new evidence that the specificity of non-clinician primary triage is limited. Alone, the primary triage is likely to lead to significant avoidable workload for urgent care providers, EDs and ambulance services owing to its poor specificity and cannot be recommended for practice as a stand-alone service. Secondary clinician triage performs an essential role in re-classifying the urgency of patients’ needs. However, our findings indicate a small risk of potential under-triage in secondary triage, associated with contextual factors such as the place, person and time at which the triage is being undertaken. The reasons for differences between services, times of the week and differences between staff undertaking secondary triage should be further investigated to understand how such differences can best be addressed through training, supervision, service design or development of the triage digital systems. These factors could also be monitored by providers, which may help inform improved operation of services.

The risk of potential under-triage is particularly present with abdominal pain, high temperature, respiratory and other common symptoms. It is also present in young children. There is a need to explore with those producing digital decision support software the scope for enhancing the accuracy of their systems in relation to these presentations, and for services to focus on the communication and IT skills essential to clinically safe triage practice. This includes the importance of clinicians giving clear, symptom-specific safety netting advice so that patients are alert to the signs of deterioration and know when to seek further urgent/emergency care. The effective management of uncertainty in acute presentations is recognised as being a crucial element of clinically safe care [31]. However, there is little empirical evidence about how safety netting advice is being implemented, including how patients experience and follow such advice. There is a need for further research to inform the training and practice of those undertaking triage and how decision support systems can support this.

## Conclusions

Our findings demonstrate the accuracy and safety of secondary triage in the context of the two-step model for urgent care triage used in England. We have previously shown that secondary triage downgrades the triage urgency in 74% of calls [5], and here we have found that this is generally achieved without risking patient safety. However, areas of potential under-triage were identified which although infrequent were mainly associated with the service and individual clinician conducting triage rather than the characteristics of the patient. Further research is needed to understand how this should be addressed.

## Electronic supplementary material

Below is the link to the electronic supplementary material.

Supplementary Material 1

## Data Availability

Data was disseminated by NHS England for this study; it is not possible to share this individual-level dataset as per the NHS England standard data sharing agreement.
