# Supplementary material for: Safety and accuracy of digitally supported primary and secondary urgent care telephone triage in England: an observational study using routine data
Source: BMC Med Inform Decis Mak. 2025 Feb 3;25:52. doi: 10.1186/s12911-025-02888-x (PMC11792721; doi:10.1186/s12911-025-02888-x)
Supplement: Supplementary file 1 — Supplementary Material 1 [file 12911_2025_2888_MOESM1_ESM.docx]

**Supplementary file**

**Further information about the digital triage software:**

**Primary triage by non-clinicians / Pathways digital triage**

When patients first call the NHS 111 telephone service in England they speak to a non-clinical operator. The operators use the Pathways digital triage tool to conduct an initial primary triage. Call operators are trained on using the Pathways tool, however they have no medical training. The Pathways tool guides the call operator to ask a series of binary questions about the patient’s symptoms, which results in triage urgency outcomes together with signposting, such as referral to the emergency service, self-care advice or referral for clinical attention. Only patients who require clinical attention are referred to an urgent care provider (a separate service providing local urgent care according to the patient’s location) for secondary triage.

**Use of Odyssey triage software in secondary triage**

Patients who are referred for secondary triage following primary triage typically receive a call back from a clinician (usually a nurse).

The clinician uses the Odyssey digital triage tool to support secondary triage. Initially, a question set is selected by the clinician based on the patient’s main presenting symptom. The Odyssey tool then presents a set of questions, each question has up to 10 pre-set answers which may be selected by the clinician. Each answer carries a weighting that contributes to the digitally recommended triage outcome, which may be one of 7 urgency levels: Emergency care, care within 1 hour, care within 2 hours, care within 4 – 6 hours, same day care (24 hours), Routine care or self-care. The clinician may override the digitally recommended urgency level, by selecting another of the other urgency levels.

One reason for this research being based on Odyssey is due to it being the only digital triage tool in England that had been accredited by the National Institute of Clinical Excellence (NICE).

**Full dataset –APC and ECDS variables provided by NHS England**

**Admitted patient care (APC) dataset.**

| ACPEND_N | Augmented Care Period End Date |
| --- | --- |
| ACPSTAR_N | Augmented Care Period Start Date |
| ADMIAGE | Age on Admission |
| ADMIDATE | Admission Date (Hospital Provider Spell) |
| ADMIMETH | Method of Admission |
| ADMISORC | Source of Admission |
| AEKEY | A&E Record Identifier |
| BEDYEAR | Bed Days Within the Year |
| CAUSE_4 | Cause Code - 4 Characters |
| DISDATE | Date of Discharge |
| DISDEST | Destination on Discharge |
| DISMETH | Method of Discharge |
| EPIDUR | Episode Duration |
| EPIEND | Episode End Date |
| EPIORDER | Episode Order |
| EPISTART | Episode Start Date |
| EPISTAT | Episode Status |
| ETHNOS | Ethnic Category |
| IMD04_DECILE | IMD Decile Group |
| INTDAYS_N | Intensive Care Level Days |
| MAINSPEF | Main Specialty |
| MYDOB | Date of Birth - Month and Year |
| OPERTN_NN | Procedure Code |
| ORGSUP_N | Number of Organ Systems Supported |
| PROCODE3 | Provider Organisation Code |
| PROCODET | Provider Code of Treatment |
| RURURB_IND | Rural / Urban Indicator |
| SEX | Current Gender of Patient |
| TRETSPEF | Treatment Specialty |

**Emergency care dataset (ECDS)**

| ACCOMMODATION_STATUS | Accommodation status |
| --- | --- |
| ACCOMMODATION_STATUS_VALID | Accommodation status valid |
| ACUITY | Urgency level assigned in ED |
| ACUITY_VALID_APPROVED | acuity valid approved |
| ARRIVAL_DATE | Date of arrival |
| ARRIVAL_MODE | Arrival Mode |
| ARRIVAL_MODE_VALID_APPROVED | arrival mode valid approved |
| ARRIVAL_TIME | arrival time |
| ASSESSMENT_DATE | assessment date |
| ASSESSMENT_TIME | assessment time |
| BIRTH_YEAR | birth year |
| CHIEF_COMPLAINT | chief complaint |
| CHIEF_COMPLAINT_EXTENDED_CODE | chief complaint extended code |
| CHIEF_COMPLAINT_VALID_APPROVED | chief complaint valid approved |
| COMORBIDITIES_NN | comorbidities |
| COMORBIDITIES_VALID_APPROVED_NN | comorbidities valid approved |
| CONCLUSION_DATE | conclusion date |
| CONCLUSION_TIME | conclusion time |
| DECIDED_TO_ADMIT_DATE | decided to admit date |
| DECIDED_TO_ADMIT_TIME | decided to admit time |
| DEPARTMENT_TYPE | department type |
| DEPARTURE_DATE | departure date |
| DEPARTURE_TIME | departure time |
| DIAGNOSIS_CODE_NN | diagnosis code |
| DIAGNOSIS_QUALIFIER_NN | diagnosis qualifier |
| DIAGNOSIS_VALID_APPROVED_NN | diagnosis valid approved |
| DISCHARGE_DESTINATION | discharge destination |
| DISCHARGE_STATUS | discharge status |
| DISCHARGE_STATUS_VALID_APPROVED | discharge status valid approved |
| INJURY_ACTIVITY_TYPE | injury activity type |
| INJURY_ACTIVITY_TYPE_VALID_APPROVED | injury activity type valid approved |
| INJURY_DATE | injury date |
| INJURY_TIME | injury time |
| INVESTIGATION_CODE_NN | investigation code |
| INVESTIGATION_DATE_NN | investigation date |
| INVESTIGATION_TIME_NN | investigation time |
| INVESTIGATIONS_VALID_APPROVED_NN | investigations valid approved |
| PREFERRED_SPOKEN_LANGUAGE | preferred spoken language |
| PREFERRED_SPOKEN_LANGUAGE_VALID | preferred spoken language valid |
| RURAL_URBAN_INDICATOR | rural urban indicator |
| SEEN_DATE | seen date |
| SEEN_TIME | seen time |
| SEQUENCE_NUMBER_NN | sequence number |
| STATED_GENDER | stated gender |
| TREATMENT_CODE_NN | treatment code |
| TREATMENT_DATE_NN | treatment date |
| TREATMENT_TIME_NN | treatment time |
| TREATMENTS_VALID_APPROVED_NN | treatments valid approved |
| TREATMENTS_VALID_APPROVED_NN TREATMENTS_VALID_APPROVED | treatments valid approved treatments valid approved |

**Result tables**

**T1 Characteristics of patients discharged within 1 day of admission**

|  | **Discharged within 1 day of admission (admitted from ED)** | **Discharged within 1 day of admission (direct admission)** |
| --- | --- | --- |
| **N calls (%)** | 3256 (54.6%) | 1447 (73.2%) |
| **Sex** | Female n= 1796 (55.2%)  Male n=1473 (44.8%) | Female n= 770 (53.2%)  Male n=677 (46.8%) |
| **Age group** |  |  |
| Infancy - under 24 months | n=617, 19.0% | n=463, 32.0% |
| 2-4 (young child) | n=277, 8.5% | n=197, 13.6% |
| 5 - 15 (child) | n=286, 8.8% | n= 176. 12.2% |
| 16-24 (young adult) | n=313, 9.6% | n=103, 7.1% |
| 25 – 34 | n=445, 13.7% | n=148, 10.2% |
| 35 – 44 | n=237, 7.3% | n=72, 4.9% |
| 44 – 54 | n=201, 6.2% | n=55, 3.8% |
| 54 – 64 | n=185, 5.7% | n=76, 5.3% |
| 64 – 74 | n=239, 7.30% | N=61, 4.2% |
| 74 – 84 | n=251, 7.70% | N=64, 4.4% |
| 85 and over | n=205, 6.30% | N=32, 2.2% |
| **Most frequent presenting symptom** | Abdominal Pain n=468, 14.4%  High Temperature n=248, 7.6%  Breathlessness n=185, 5.7%  Cough n=168, 5.2%  Chest Pain n=154, 4.7%  Vomiting n=140, 4.3%  Rash n=123, 3.8%  Back pain n=92, 2.8%  Unwell (no specific symptom specified), n=76, 2.3%  Headache n=71, 2.2% | Abdominal Pain n= 197, 13.6%  High Temperature n= 194, 13.4%  Cough n=120, 8.3%  Rash n=89, 6.1%  Vomiting n=73, 5.0%  Diarrhoea n=46, 3.2%  Breathlessness n=42, 2.9%  Unwell (no specific symptom specified) n=33, 2.3%  Back pain n=32, 2.2%  Sore throat n=29, 2.0% |

**T2: Percentages, sensitivity, specificity, and positive/negative predictive values of primary and secondary triage levels for patients attending ED within 24 hours.**

| PRIMARY TRIAGE* URGENCY LEVEL | Number of calls | did not attend | n attended | % attended | Sensitivity | Specificity | PPV | NPV |
| --- | --- | --- | --- | --- | --- | --- | --- | --- |
| Emergency | n/a | n/a | n/a | n/a | n/a | n/a | n/a |  |
| Care within 1 hour | 36,485 | 27,743 | 8,742 | 24.0% | 46.6% | 65.4% | 24.0% | n/a |
| Care within 2 hours | 33,943 | 28,090 | 5,853 | 17.3% | 77.8% | 30.4% | 20.7% | 76.0% |
| Care within 4-6 hours | 15,822 | 12,878 | 2,944 | 18.6% | 93.5% | 14.3% | 20.3% | 79.3% |
| Same day care (<24 hours) | 11,296 | 10,187 | 1,109 | 9.8% | 99.4% | 1.6% | 19.1% | 79.7% |
| Routine care | 358 | 314 | 44 | 11.7% | 99.6% | 1.2% | 19.1% | 80.9% |
| Self-care/no urgency | 1,042 | 965 | 77 | 7.1% | 100.0% | 0.0% | 19.0% | 80.9% |
| Total | 98,946 | 80,177 | 18,769 | 19.0% |  |  |  |  |
| SECONDARY TRIAGE** URGENCY LEVEL |  |  |  |  |  |  |  |  |
| Emergency | 1,966 | 625 | 1,341 | 68.4% | 7.2% | 99.2% | 68.2% | n/a |
| Care within 1 hour | 3,636 | 1,192 | 2,444 | 67.5% | 20.2% | 97.7% | 67.6% | 31.8% |
| Care within 2 hours | 14,382 | 8,982 | 5,400 | 37.5% | 48.9% | 86.2% | 46.0% | 32.4% |
| Care within 4-6 hours | 37,758 | 31,868 | 5,890 | 15.6% | 80.4% | 45.3% | 26.1% | 54.0% |
| Same day care (<24 hours) | 11,493 | 10,136 | 1,357 | 11.7% | 87.6% | 32.3% | 23.7% | 73.9% |
| Routine care | 11,084 | 10,127 | 957 | 8.7% | 92.7% | 19.3% | 21.6% | 76.3% |
| Self-care/no urgency | 18,424 | 17,054 | 1,370 | 7.4% | 100.0% | 0.0% | 19.0% | 78.4% |
| Total | **96,777** | **78,003** | **18,774** | 19.4% |  |  |  |  |

| *P value <0.001 for differences in percentages attending, across primary triage urgency levels (chi squared test for proportions) |
| --- |
| ** P value <0.001 for differences in percentages attending, across secondary triage urgency levels (chi squared test for proportions) |

**T3: Percentages, sensitivity, specificity, and positive/negative predictive values of primary and secondary triage for patients admitted to inpatient care within 24 hours.**

| PRIMARY TRIAGE URGENCY LEVEL | Number of calls | Not admitted | n admitted | % admitted | Sensitivity | Specificity | PPV | NPV |
| --- | --- | --- | --- | --- | --- | --- | --- | --- |
| Emergency | n/a | n/a | n/a | n/a | n/a | n/a | n/a | n/a |
| Care within 1 hour | 36,485 | 32,909 | 3,576 | 9.8% | 45.1% | 63.8% | 9.8% | n/a |
| Care within 2 hours | 33,943 | 31,016 | 2,927 | 8.6% | 82.0% | 29.8% | 9.2% | 90.2% |
| Care within 4-6 hours | 15,822 | 14,861 | 961 | 6.1% | 94.1% | 13.4% | 8.7% | 90.8% |
| Same day care (<24 hours) | 11,296 | 10,880 | 416 | 3.7% | 99.4% | 1.5% | 8.1% | 91.3% |
| Routine care | 358 | 341 | 17 | 4.7% | 99.6% | 1.1% | 8.1% | 91.9% |
| Self-care/no urgency | 1,042 | 1,008 | 34 | 3.3% | 100.0% | 0.0% | 8.0% | 91.9% |
| Total | 98,946 | 91,015 | 7,931 | 8.0% |  |  |  |  |
| SECONDARY TRIAGE  URGENCY LEVEL |  |  |  |  |  |  |  |  |
| Emergency | 1,966 | 1,477 | 489 | 24.9% | 6.2% | 98.4% | 24.9% | n/a |
| Care within 1 hour | 3,636 | 2,808 | 828 | 22.8% | 16.6% | 95.3% | 23.5% | 75.1% |
| Care within 2 hours | 14,382 | 12,107 | 2,275 | 15.8% | 45.3% | 82.0% | 18.0% | 76.5% |
| Care within 4-6 hours | 37,758 | 34,896 | 2,862 | 7.6% | 81.5% | 43.5% | 11.2% | 82.0% |
| Same day care (<24 hours) | 11,493 | 10,971 | 522 | 4.5% | 88.1% | 31.4% | 10.1% | 88.8% |
| Routine care | 11,084 | 10,696 | 388 | 3.5% | 93.0% | 19.7% | 9.2% | 89.9% |
| Self-care/no urgency | 18,424 | 17,866 | 558 | 3.0% | 100.0% | 0.0% | 8.0% | 90.8% |
| Total | 98,743 | 90,821 | 7,922 | 8.0% |  |  |  |  |

| *P value <0.001 for differences in percentages admitted, across primary triage urgency levels (chi squared test for proportions) |
| --- |
| ** P value <0.001 for differences in percentages admitted, across secondary triage urgency levels (chi squared test for proportions) |

**T4 Characteristics of admitted patients (n = 246), which had been potentially under-triaged, by age**

| Age group | N | Percentage |
| --- | --- | --- |
| Infancy - under 24 months | 53 | 21.5 |
| 2-4 (young child) | 28 | 11.4 |
| 5 - 15 (child) | 15 | 6.1 |
| 16-24 (young adult) | 18 | 7.3 |
| 25 – 34 | 20 | 8.1 |
| 35 – 44 | 9 | 3.7 |
| 45 – 54 | 18 | 7.3 |
| 55 – 64 | 21 | 8.5 |
| 65 – 74 | 22 | 8.9 |
| 75 – 84 | 21 | 8.5 |
| 85 and over | 21 | 8.5 |
| Total | 246 | 100 |

**T5 Characteristics of admitted patients (n = 246), which had been potentially under-triaged, by sex**

| Sex | N | Percentage |
| --- | --- | --- |
| Female | 120 | 48.8 |
| Male | 126 | 51.2 |
| Total | 246 | 100 |

**T6 Characteristics of admitted patients (n = 246), which had been potentially under-triaged (Top 10 symptoms)**

| Top 10 main symptoms | N | Percentage |
| --- | --- | --- |
| High Temperature | 31 | 12.6 |
| Cough | 29 | 11.8 |
| Abdominal Pain | 23 | 9.4 |
| Breathlessness | 23 | 9.4 |
| Rash | 12 | 4.9 |
| Unwell | 10 | 4.1 |
| Urinary Symptoms | 10 | 4.1 |
| Vomiting | 10 | 4.1 |
| Cold/Flu | 7 | 2.9 |
| Constipation | 6 | 2.4 |

**T7 Characteristics of admitted patients (n = 1477), which had been potentially under-triaged, by age**

| Age group | N | Percentage |
| --- | --- | --- |
| Infancy - under 24 months | 299 | 20.24 |
| 2-4 (young child) | 118 | 7.99 |
| 5 - 15 (child) | 126 | 8.53 |
| 16-24 (young adult) | 115 | 7.79 |
| 25 – 34 | 151 | 10.22 |
| 35 – 44 | 94 | 6.36 |
| 45 – 54 | 97 | 6.57 |
| 55 – 64 | 103 | 6.97 |
| 65 – 74 | 122 | 8.26 |
| 75 – 84 | 137 | 9.28 |
| 85 and over | 115 | 7.79 |
| Total | 1,477 | 100 |

**T8 Characteristics of admitted patients (n = 1477), which had been potentially under-triaged, by sex**

| Sex | N | Percentage |
| --- | --- | --- |
| Female | 791 | 53.55 |
| Male | 686 | 46.45 |
| Total | 1,477 | 100 |

**T9 Characteristics of admitted patients (n = 1477), which had been potentially under-triaged (top 10 symptoms).**

| Top 10 main symptoms | N | Percentage |
| --- | --- | --- |
| Abdominal Pain | 199 | 13.47 |
| High Temperature | 147 | 9.95 |
| Cough | 97 | 6.57 |
| Vomiting | 70 | 4.74 |
| Rash | 53 | 3.59 |
| Breathlessness | 47 | 3.18 |
| Unwell | 47 | 3.18 |
| Urinary Symptoms | 46 | 3.11 |
| Diarrhoea | 42 | 2.84 |
| Back pain | 40 | 2.71 |
